# Machine learning algorithms distinguish discrete digital emotional fingerprints for web pages related to back pain

**DOI:** 10.1038/s41598-023-31741-2

**Published:** 2023-03-21

**Authors:** Davide Caldo, Silvia Bologna, Luana Conte, Muhammad Saad Amin, Luca Anselma, Valerio Basile, Md. Murad Hossain, Alessandro Mazzei, Paolo Heritier, Riccardo Ferracini, Elizaveta Kon, Giorgio De Nunzio

**Affiliations:** 1 Humanitas Gradenigo Hospital, Turin, Italy; 2 Imparamare Ong, Asti, Italy; 3 Mathematics and Physics Department “Ennio de Giorgi”, University of Salento, Lecce, Italy; 4 Informatic Department, Turin University, Turin, Italy; 5 Digspes Department, Oriental Piedmont University, Alessandria, Italy; 6 Genoa University, Genoa, Italy; 7 IRCCS Humanitas Research Hospital, Milan, Italy

**Keywords:** Quality of life, Diseases, Medical research, Health care

## Abstract

Back pain is the leading cause of disability worldwide. Its emergence relates not only to the musculoskeletal degeneration biological substrate but also to psychosocial factors; emotional components play a pivotal role. In modern society, people are significantly informed by the Internet; in turn, they contribute social validation to a “successful” digital information subset in a dynamic interplay. The Affective component of medical pages has not been previously investigated, a significant gap in knowledge since they represent a critical biopsychosocial feature. We tested the hypothesis that successful pages related to spine pathology embed a consistent emotional pattern, allowing discrimination from a control group. The pool of web pages related to spine or hip/knee pathology was automatically selected by relevance and popularity and submitted to automated sentiment analysis to generate emotional patterns. Machine Learning (ML) algorithms were trained to predict page original topics from patterns with binary classification. ML showed high discrimination accuracy; disgust emerged as a discriminating emotion. The findings suggest that the digital affective “successful content” (collective consciousness) integrates patients’ biopsychosocial ecosystem, with potential implications for the emergence of chronic pain, and the endorsement of health-relevant specific behaviors. Awareness of such effects raises practical and ethical issues for health information providers.

## Introduction

Degenerative musculoskeletal chronic pain is a very significant and costly problem throughout the industrialized world with low back pain being the leading cause of disability^1^. The current concept of treatment applies when the local anatomy is macroscopically altered as demonstrated by imaging and alterations become an established source of chronic pain; treatments largely target the organic substrate and include analgesic drugs and surgical decompression or fusion^2^. Current approaches showed major limits: systematic reviews find scant evidence of medication efficacy^3^ and diffuse severe complications following opioid widespread use^4^.

Musculoskeletal chronic pain is also known to be related to disease-specific psychological and social components, intertwined in a variety of proportions with the organic substrate, for instance: fibromyalgia is mostly driven by central nervous predispositions^5^; hip and knee osteoarthritis have a larger peripheral nociceptor contribution, also driving a better success rate of pain relief with joint replacement surgery^6^; as high as 90% of back pain cases are classed as non-specific, as there is often no definite organic substrate cause for the experienced pain, putting it somewhere in between the fibromyalgia and the great joint degeneration end of the spectrum of musculoskeletal degenerative diseases^7^. Failed back surgery rates 20–40% resulting in persistent pain^8^.

Specific emotional patterns emerged to relate to different musculoskeletal degenerative diseases^9–17^; the emotional content of external sources of information can complement rather than mirror users’, in a non-straightforward complex system, in analogy with the counterintuitive role of sadness in the pleasure elicited by sad music^18^. Received information acts as a countering or favoring agent for the emergence of chronic pain in the biopsychosocial arena^19^. Affective content plays an integrated role in the process^20^.

The dominant source of information for patients in modern society is the internet^21^. The success of internet pages is the result of a dynamic interplay between users that “socially validate” selected pages (by visualization, interaction, and other actions) and search engine algorithms that amplify the visibility of such validated pages, raising them toward the top search output listed pages; further social validation is thus acquired and a phenomenon of self-increased popularity is engaged; the final results is that a small fraction of high ranked pages inform the great majority of users^22^.

Sentiment analysis (also known as opinion mining) is the systematic identification, extraction, quantification, and study of affective states using natural language processing, text analysis, and computational linguistics via computer science^23^.

Machine Learning (ML) can analyze large amounts of data and perform classification with measurable predictivity^24^.

On such premises, we formulated the general hypothesis that “successful” internet pages related to a given pathology can trigger their own validation by users by embedding a specific, consistent emotional pattern, complementary to patients’ biopsychosocial ecosystem.

Thus, supervised ML algorithms would accurately discriminate the topic of the original text by the affective emotional fingerprint produced by sentiment analysis. In the present research top ranking English language websites are analyzed, comparing two pooled conditions: the first one is the “nonspecific” degenerative chronic lumbar back pain (LBP), characterized by a cluster of multiple organic substrate alterations variably combined and highly non-specifical, leading to high rates of failed surgical treatment; the other is great joint (hip and knee) chronic degenerative disease, with more specific substrate alteration, generally leading to the higher success rate of surgical treatment.

The scope of our work is to test whether a consistent affective pattern characterizes the corpus of knowledge socially validated and related to a specific pathology, allowing discrimination from a control group characterized by a different relative specific weight of biopsychosocial components. In particular, the presence of discriminating emotions would be consistent with somatic marker theory applied to the emotional domain in medicine^20^. Such a result would suggest a specific role for emotional fingerprints. As far as the authors know there is no previous systematic analysis of the affective content of medical internet pages concerning low back pain and other musculoskeletal degenerative diseases. This is a relevant gap of knowledge since digital emotional content integrates the patient’s biopsychosocial system, with a potential role in the emergence of chronic pain and enforcing/blocking positive/negative social behaviors; major relevance implications would arise for institutions responsible for the diffusion of healthcare-relevant information and in general for medical information providers on the internet both on the practical and ethical plane. If the hypothesis were verified, ethical considerations related to the largely subliminal effects of emotional elements embedded in website information would need further discussion by scientific and policy-making representatives.

No humans were involved in the study, which is based on data openly available from the authors upon reasonable request.

The paper is structured as follows. In “Literature review” we discuss the studies that constitute the background of the study. In “Results” we introduce data description and data analysis results. In “Discussion” we comment the results and provide some possible interpretations suggesting further implications. Finally, in “Materials and methods” we describe the data selection process, the identification of Internet resources, and the data analysis process.

## Literature review

The notion of information being a key factor in health/disease emergence is in general consistent with the Biopsychosocial (BPS) model that suggests that a person's state of wellness or illness is not coincident with the organic substrate alteration but rather intertwined with psychological and social factors^25^. The critical role of Emotions, outlined by decades of affective neuroscience findings, tightly integrates affective domains in the model^20^. Emotions ultimately bias or determine behavior according to the somatic marker theory^26,27^. In fact, the emergence of chronic pain in neuroscientific literature has been linked via functional neuroimaging to the activation of several sensorial, cognitive, and affective central nervous system circuits^28^. The main BPS model limit remains the lack of tools for clinical usability: thus, the heuristic value of a reductionistic “mechanical” approach largely prevailed in clinical settings^29^. The BPS model evolved by incorporating notions from Ashby's law of requisite variety, Rothman's notion of multiple sufficient causes of a condition, and top-down causation in complex adaptive systems^30^. Within such a theoretical frame degenerative musculoskeletal chronic pain can be interpreted as an emergent property of a complex adaptive system, with affective information being a critical element of the system^19,30^.

LBP is the main source of chronic pain and disability in the world^1^, linked in a controversial way with emotional regulation, somatosensory amplification, and rumination in negative affective or dysfunctional beliefs^10^. LBP psychological arena more frequently includes fear^11^, anger, and sadness in a frame of catastrophism, anxiety, or depression^12^. Fear has a direct effect on the outcome of patients, influencing behavior: fear of pain and/or injury/movement leads to movement avoidance, and it is possibly implicated in the transition from acute to chronic and the persistence of disabling LBP^31^. Anger is another leading emotion in many LBP studies, with greater effects on chronic pain severity than sadness: it is shown that people who tend to express anger and who exhibit high pain sensitivity could be characterized by deficits in endogenous inhibitory mechanisms^32^. A symptom-specific reactivity model showed that anger arousal may lead to increases in muscle tension near the site of injury, and thereby increase pain; increases in lower paraspinal muscle tension are higher in anger than in sadness, and patients with elevated anger expressiveness showed greater increases in muscle tension^13^.

The estimated 2010 prevalence of total hip and total knee replacement among the total US population was 0.83% and 1.52%, respectively^33^. Chronic pain despite joint replacement is not uncommon, affecting approximately 10% of patients after total hip replacement and 20% of patients after total knee replacement^34^. The related emotion reported in scientific literature is fear^14^, altogether with withdrawal and depression^15^. Psychological and structural factors interact exacerbating pain perception^16,17^.

A characteristic of people with chronic pain is avoidance: the “cognitive-behavioral fear-avoidance model” includes cognitive (idiosyncratic maladaptive beliefs on pain), affective (fear), and behavioral (avoidance) components^35^.

Disgust is one of the basic emotions characterized by a strong sense of aversion, associated with physical reactions (nausea, sweating and lowering blood pressure)^36^. It is an emotion of well-rooted evolutionary origin: animals have evolved a series of behaviors to reduce the risk of infection by pathogens, such as microorganisms^37^. Disgust showed the tendency to lead to a complex regulation of immune-related functions, effects similar to the acute phase response to infections; immediately after a disgust induction, reported pain is reduced, but later it increases leading to a final higher pain sensitivity^36^.

Although disgust was first thought to be a motivation for humans to avoid only physical contaminants, it has since been applied to psychosocial contaminants as well. Likewise, when a group experiences someone who commits violence or misdemeanor to another member of the group, its reaction is to “divert” that person from the group, basically the same recorded when contaminating fluids are involved^38^. When one experiences disgust, this emotion might signal that certain behaviors, objects or people are to be avoided in order to preserve psychosocial purity^39^, as opposed to other emotions such as fear, anger, and sadness that appear "unrelated to moral judgments of purity". The emotion of disgust can be hypothesized to serve as an effective mechanism following occurrences of negative social value provoking repulsion and desire for social distance. The origin of disgust can be defined by motivating the avoidance of offensive behavior, and in the context of a social environment, it can become an instrument of social avoidance. Disgust is known to reduce motivations for social interaction^40^. Social interactions are a key component of well-being in the aging population, with the growth of emotional empathy serving as a compensation factor to cognitive age-related decadence of cognitive empathy^41^.

Several studies have shown that the internet (through social media) can be a representative source of data, exploitable to recognize public perceptions and behaviors during a crisis, and even predict outbreaks^42,43^. In modern society, information is increasingly being sought on the internet^44^. In the medical field, its relevance has grown rapidly: in the United States, for example, the number of people seeking medical information on the Internet has increased from 54 million in 1998 to about 117 million in 2005^45^. In January 2022, 4.66 billion people accessed the internet; GWI’s survey also finds that 25.9 percent of working-age internet users check health symptoms online every week^44^.

ML algorithms showed predictive accuracy in many biology, medicine, and even social system applications; precision medicine in the twenty-first century strives for accurate prediction of what is beneficial for individual patients; prediction, as opposed to association, comes into play when forecasting outcomes that are yet unobserved. Nonetheless, relevance is not a synonym for discriminant power as used in classification and prediction: significant variables in a statistical model do not guarantee prediction performance, and non-significant attributes might reveal predictive^46^. Machine learning algorithms have been previously applied to the field of pain affection^47^.

An entirely new discipline called Social Neuroscience is based on neurophysiological (i.e. mirror neuron and internal simulation activity) and social evidence of the notion of collective consciousness (CC), the set of shared knowledge, assumptions, moral attitudes operating as a unifying force within society^48^; CC includes collective emotions, emergent macrolevel affective processes that cannot be readily captured at the individual level^49^. The CC “storing” substrate evolved in time: by the end of the eighties of the twentieth century less than 1% of the world’s information was archived in a digital format, whereas in 2007 this percentage reached 94% and rapidly growing^21^. The original concept of a Virtual Collective Consciousness derived from humanities and was initially restricted to social networking influencing behavior^50,51^. The tight relation of CC with digital information is the result of a dynamic interplay between users and search engine algorithms: users determine “social validation” of web pages by interacting with the ones best complementing their psychological drive; search engine algorithms (like Google’s PageRank) further enhance visibility of those pages putting them at the top of the search engine query results, closing a self-powered circuit for the constitution of a pool of “successful” and widely accessed digital information^22^. The digital information feeds the cognitive and affective knowledge of patient collectivity: it has been proposed that multiple people can converge on the same emotion pattern when exposed to digital media^52^ and that the BPS model must evolve to include a “digital” component, developing a ‘biopsychosocial-digital’ approach to health^53^.

## Results

### Relevant sources identification

We a selection of an arbitrary number of 2000 sites in English, French, German, Italian and Spanish with high relevance/popularity for the conditions of interest. The sites were listed in an Excel diagram. We then identified 837 Sites in English. It was plotted the qualitative fingerprint of all the different languages; the English language pages were forwarded to quantitative analysis for greater consistency in the result, to exclude variables related to linguistic influence that may be the subject of subsequent studies. The analyzed sites concerned the following conditions of musculoskeletal pathology: back pain, herniated disc, hip prosthesis, knee prosthesis, low back pain. Within the 837 English sites the various pathologies were distributed as illustrated in Table 1.

**Table 1:** Number of documents considered for each pathology (English sites).

Label	Count
Back pain	165
Disk herniation	176
Low back pain	164
Hip prosthesis	168
Knee prosthesis	164

### Semantic analysis

**Figure 1:**
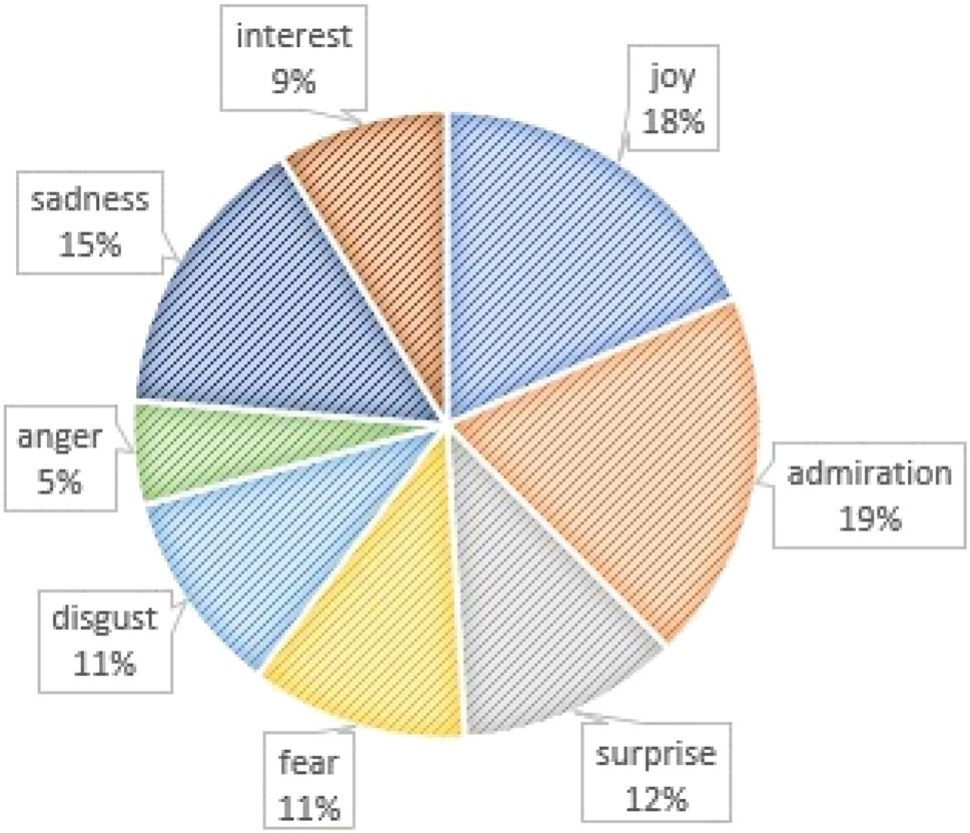
Prevalence of emotion words in all selected sites.

**Figure 2:**
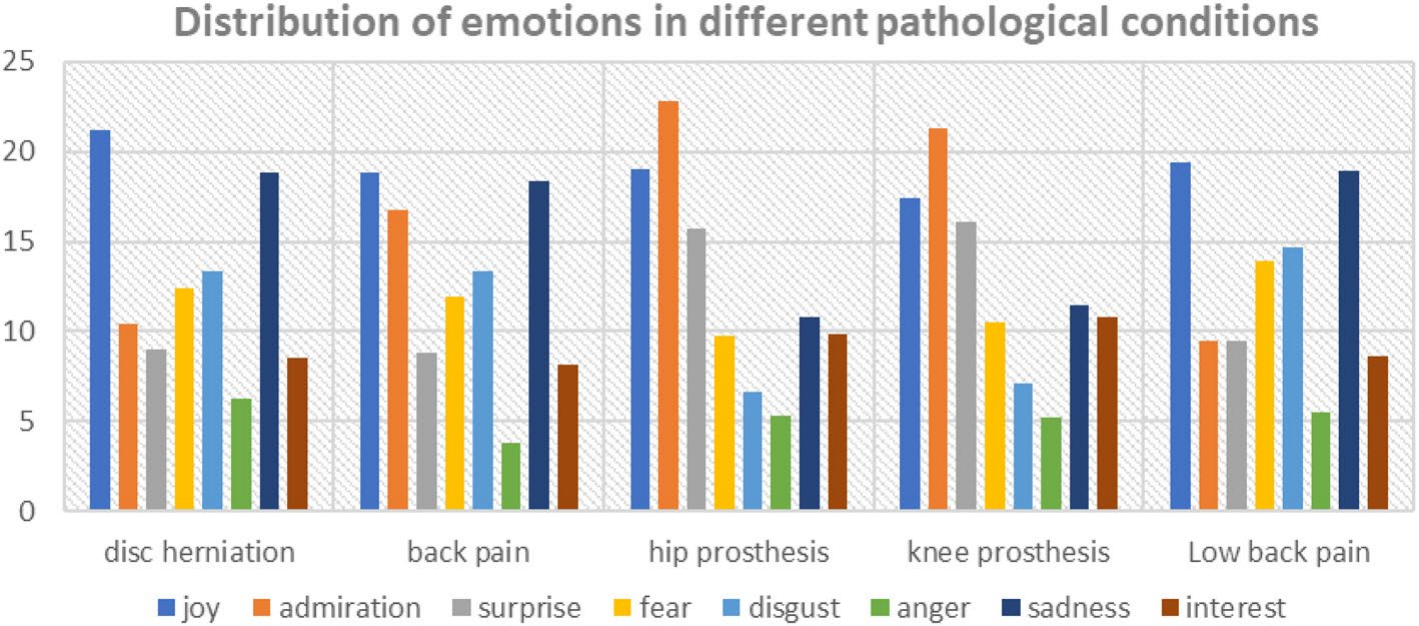
Charts illustrating prevalence of emotion words in different conditions.

Emotion content word distribution, normalized to number of emotion words per site, is depicted in Fig. 1. Distribution of emotion content words per condition and per emotion is illustrated in Fig. 2.

**Figure 3:**
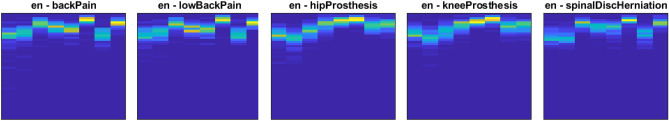
Emotional fingerprints for the documents related to the five health conditions considered (English language). In the fingerprint, eight “pixel” columns left to right refer to joy, admiration, etc., and the intensity of the emotion (in arbitrary scale) increases top to bottom.

The qualitative analysis leads to a data visual pattern: the emotional fingerprints in Fig. 3. The fingerprints are substantially histograms of per-document (scaled) counts of emotional content words: each histogram occupies a column in the image, with bins arranged top to bottom and refers to the one of the considered emotions, so each fingerprint contains 8 hystograms. Warm colors indicate highly occupied bins while cold colors are empty bins.

### Nonparametric statistical tests and machine learning

We decided to compare the subsets of texts for the different health conditions in a pairwise fashion, both graphically with scatterplots, and computationally with significance tests (Mann–Whitney U-test) and a ML approach, to assess if the sets of documents significantly differed at group level and at individual level.

The internet documents were modeled as vectors of variables (the emotional scores for joy, admiration, surprise, fear, disgust, anger, sadness, and interest) and labeled by the health conditions of interest (“classes”).

**Figure 4:**
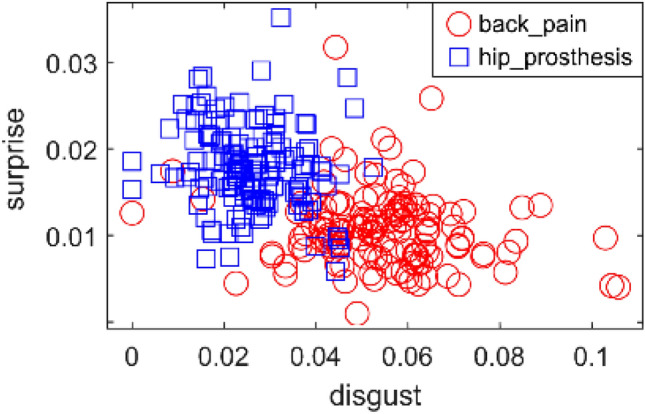
Scatterplot showing the distributions of the Emotional score variables related to disgust and surprise, for back pain vs hip prosthesis documents (English language).

Some variables (in particular, disgust) showed a large discriminating power. Some pairs of variables, too, were discriminating when considered together (e.g., disgust and surprise). A few features were quite strongly correlated, such as disgust and sadness. Figure 4 shows the example case of scatterplot for English language, back pain vs hip prosthesis documents. In this example, surprise and disgust are the two paired emotions and their peculiar distributions are signals of important discriminating power.

Statistical nonparametric tests (Mann–Whitney U-test) applied to assess univariate emotional score group differences between documents for different health conditions, mostly failed in finding low p-values (which would allow to reject the null hypothesis that the documents from two classes come from the same distribution). On the contrary, ML employed to classify the documents detected many cases with large classification accuracy, which proves that document emotional contents have peculiar patterns in each class of a pair of health conditions, making them different and recognizable. The accuracy and other statistics for the English-language documents were calculated for all the pairs of conditions of interest, as shown in Table 2 which reports the interesting cases with accuracy > 0.9 for linear Support Vector Machine (SVM) classification.

**Table 2:** The columns report various classification statistics of a linear SVM classifier trained and validated with a fivefold cross-validation scheme for the English language and for the discrimination between various health conditions in pairs (cases with accuracy > 0.9 were selected); 2nd to 7th columns contain the confusion matrix [*true positives, false positives; false negatives, true negatives*], sensitivity, specificity, precision, accuracy, and the F1 score. The last column shows the p-values issued by the Mann–Whitney U-test.

Support vector machine classifier	Confusion matrix	Recall or sensitivity	Specificity	Precision	Accuracy	F1 Score	Mann–Whitney U-test p-value
Back pain vs hip prosthesis	[159, 6; 8, 160]	0.95	0.96	0.96	0.96	0.96	0.48
Back pain vs knee prosthesis	[160, 5; 8, 156]	0.95	0.97	0.97	0.96	0.96	0.049
Disc herniation vs knee prosthesis	[165, 11; 10, 154]	0.94	0.93	0.94	0.94	0.94	0.96
Disc herniation vs hip prosthesis	[162, 14; 11, 157]	0.94	0.92	0.92	0.93	0.93	0.062
LBP vs hip prosthesis	[156, 12; 10, 154]	0.94	0.93	0.93	0.93	0.93	1.2
LBP vs knee prosthesis	[155, 9; 6, 158]	0.96	0.95	0.95	0.95	0.95	0.28

**Figure 5:**
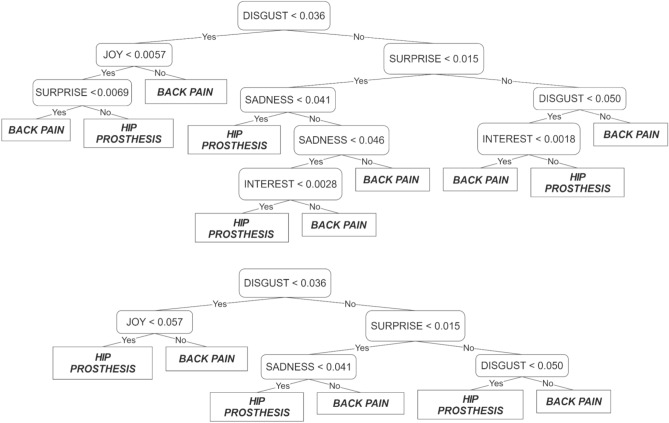
Decision tree before and after pruning, for the English language, back pain vs hip prosthesis.

**Figure 6:**
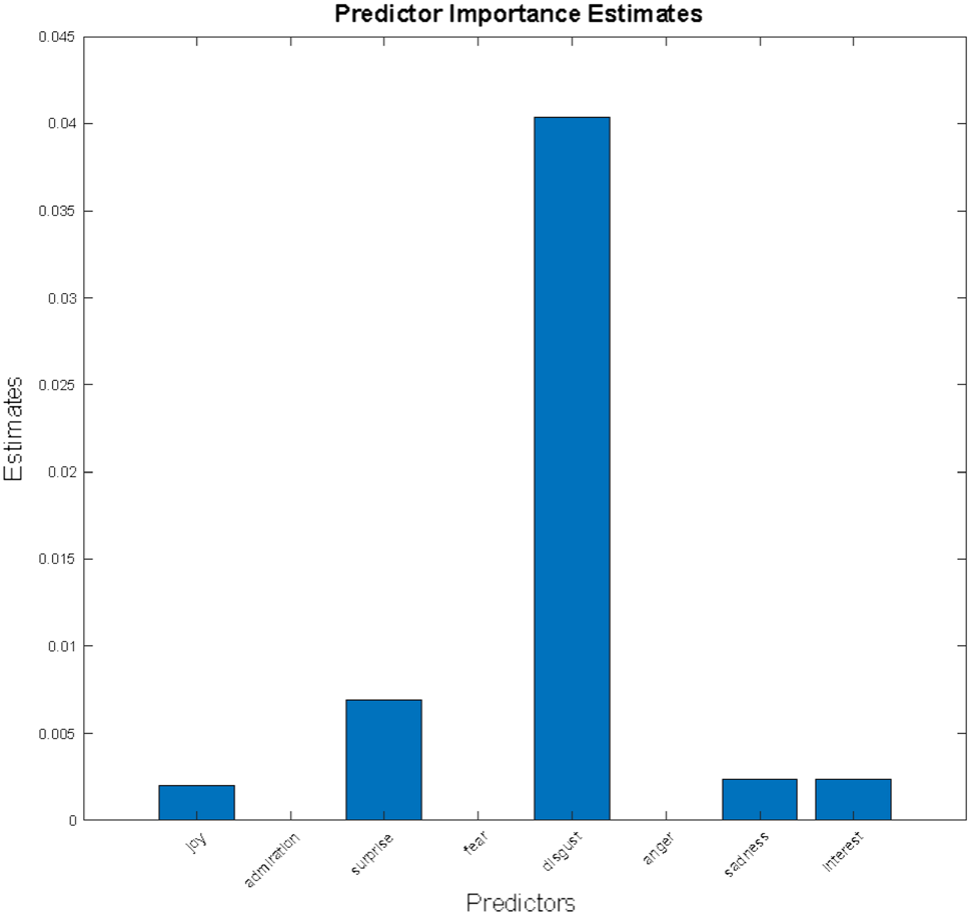
Estimate of predictor importance, for English language, back pain vs hip prosthesis.

Using decision trees instead of SVMs gave very similar results and allowed us to explore the role of the emotion variables. The graphics in Fig. 5 show the decision tree before and after pruning. Pruning is a tree compression technique that removes sections of the decision tree that are redundant and have little influence on classification accuracy. Pruning decreases classifier complexity and has two consequences: it reduces overfitting (possibly enhancing generalization) and improves explainability. Figure 6 shows the estimates of predictor importance, consistent with the trees.

## Discussion

To give back depth to pain experience in musculoskeletal degenerative pathology it is necessary to reinterpret it within the BPS approach and consider its information source feeds with specific attention to the emotional component. Digitalization shifted from an irrelevant share to nearly the totality of the health knowledge corpus, coupled with widespread access to the internet, dramatically changing the scenario of healthcare information. The described reciprocal interaction between the users and the affective content of digital medical information selects a highly representative sub-corpus of information: for practical reasons, it is proposed to be referred to as “digital affective collective consciousness” (DACC). The DACC includes the affective subset of the “virtual collective consciousness'', embedded in successful pages related to every area of human knowledge. Sentiment analysis is a useful method to characterize DACC projection to entire sectors of information or specific topics on the internet, relating to a basic emotion model; such assessment is extremely relevant when pathologies with a major psychosocial content are investigated. LBP generally does not relate to a specific major organic lesion, but rather the result of a combination of many minor degenerative lesions with a “non-specificity” that parallels fibromyalgia, and a major psychosocial component, including a vast array of emotions. Emotions ultimately bias or determine behavior according to the somatic marker theory according to somatic marker theory^26,27^.

We identified substantial histograms of per-document (scaled) emotional word counts as an easy and novel graphical way to plot quantitative presence of emotion-related words with their intensity in the emotional scale.

From our digital information analysis, major relevance for disgust emerged as a key discriminating factor between LBP and hip/knee affection-related internet pages; disgust acts as the first bifurcations in all the ML decision trees generated (see “Materials and methods” and “Results”); in some cases, the degree of disgust alone identifies the original topic from the affective pattern; in other cases, ML relies on a combination of disgust with surprise, joy or sadness. The difference in disgust intensity in the two affective patterns of spine and control pathology may reflect the different biopsychosocial profile of affections.

Disgust is a diverging emotion: it would not be expected to be associated with popularity. It can be argued that digital information of disgust could be involved in the “adaptive” characteristic of the pain system, the “diverting from disease” impulse endorsing avoidance strategy. It can be conjectured that the temporary decrease in pain sensitivity plays a role in internet pages' success. If that is the case, the later sensitization to chronic pain may contribute to a net negative outcome. Such a scenario calls for specific research since it raises serious concerns about digital information subliminal emotional long-term effects; scientific, but also ethical, and legal implications are raised.

In general, the role of disgust may relate to different ways of perceiving some characteristics intrinsic to each of the two groups of affections.

Differences in the emotional profile of the patients have been addressed in the Literary Review section. Furthermore, spine and great joint degenerative conditions both affect walking capability, but with different pathways^54^. This may be a potential source of the psychosocial divide between the two groups of conditions, reflected in the pages promoted in the internet user-algorithm dynamic; in the structure of disgust motion is less relevant compared to other emotions such as fear, since disgust can relate to disembodied entities, such as moral judgment.

The component of attraction power detailed by Sachs^18^ is based on the pure aesthetic power of the art; in that sense diverting emotions such as sadness or disgust could be felt on its aesthetic value alone. The authors are not aware of any published study concerning the assessment of the intrinsic aesthetic feature and role of medical websites, in particular concerning pages related to spine, hip, or knee pathologies.

Some limits of the present work are acknowledged. The control group was chosen arbitrarily; the decision, though, was based on a similar condition from a medical perspective (concerning epidemiology, timing, and roadmap of treatment) to elude potential hidden variables, but with the characterizing differences of the surgical outcome. In our results statistical association did not match predictivity; it has been shown the latter represents the best approach to complement explanatory models and setting new neuroscientific theoretical grounding^24^, in our work we combined the explanatory power of the somatic marker theory with the predictive power of modern computational techniques to overcome limitations of mere statistical association, namely ML algorithms to predict the pathology from the emotional content of the page. Furthermore, we extended the implications of somatic market theory to social neuroscience in the arena of the digital information society. This could pave the road not only to a deeper understanding of biopsychosocial pathologies but also to an updated version of the bps model. Also, we point out that the fingerprint methodology is a novel representational construct of emotion representation, particularly fitting for comparative tests (in our case, an intra-linguistic one).

More tests need to be performed to fully outline the extent and generalize the relevance of the findings, laying the ground for subsequent potential applications. Quantitative analysis was limited to the English language only to contain language-dependent biases. The study of fingerprints (Fig. 3) may be taken up in later studies for inter-language comparison. Only the presence of words attributed to specific emotions is considered by Senticnet; more advanced methods also consider text complexities such as negation or sarcasm, or emotional word valence that can change according to context and domain, although several works adopt this simplified methodology which in practice can work adequately^55^.

In conclusion, ML predictivity shows a specific emotion pattern strictly related to a specific medical condition when “successful” medical web pages are concerned; in the case of the study, disgust was shown to be the single basic emotion with more relevant discriminative power between the spine and great joint degenerative conditions, outlining a different psychosocial profile. Different explanatory hypotheses have been proposed, to be investigated in future research. The notion of DACC is outlined as a conceptual framework. Future research should consider other languages for comparison, sentiment analysis of digital information produced by patients, i.e., through social networks, or confrontation of digital emotional content to real-life emotional frames; they may also focus on other sectors of DACC and pathologies, possibly leading to new behavioral analysis models. Exploiting such knowledge holds the potential to overcome limitations of treatments based on mechanistic pathogenetic reductionism, enforcing a modern BPS model that includes DACC role; the process may lead to a more comprehensive understanding of how internet information spreads, drive application of modern neuroscience emerging evidence to medical practice, optimizing approaches to major medicinal issues, pursue correct information to the public, ultimately leading to optimization of cures and greater prosperity of the community.

## Material and methods

### Identifying relevant sources

**Figure 7:**
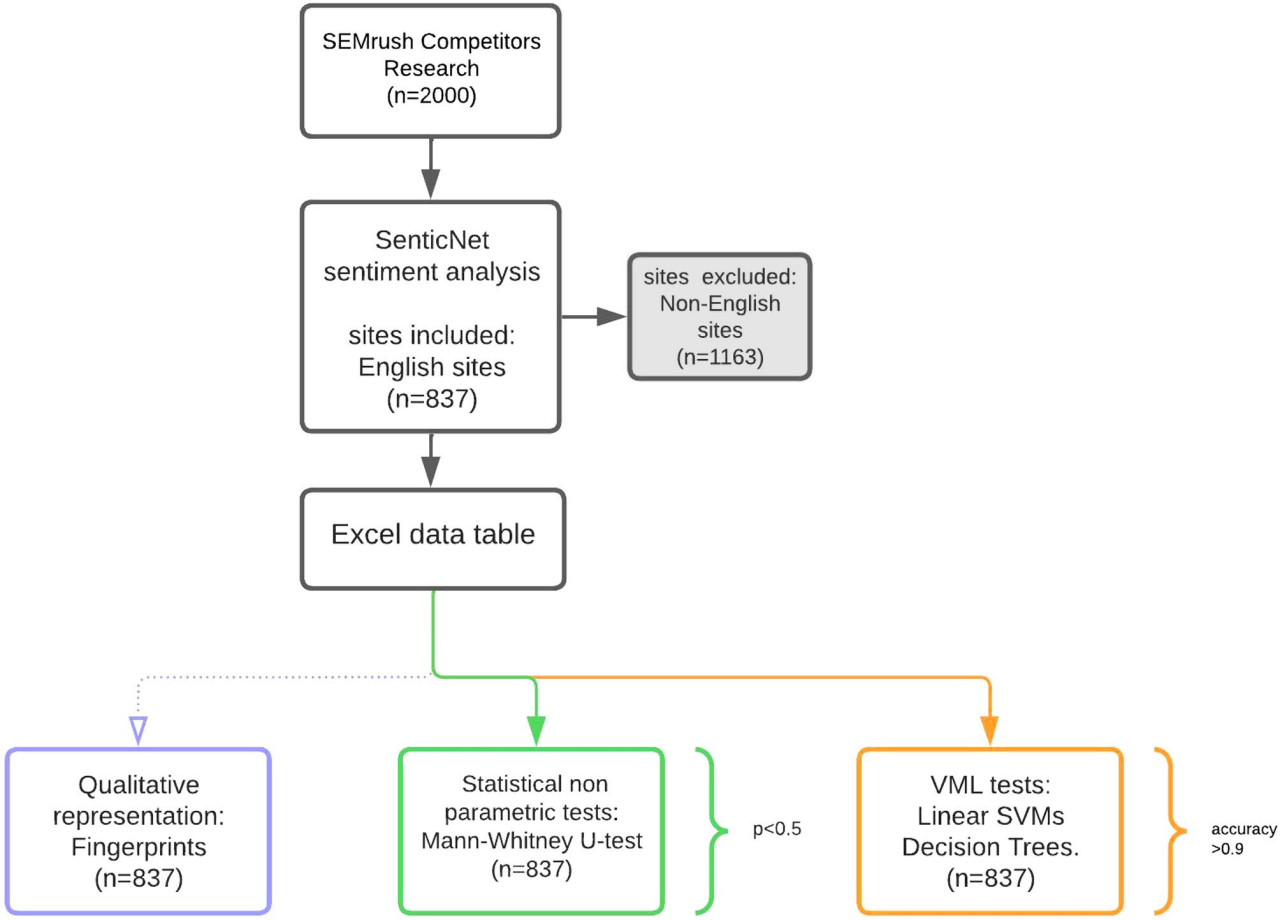
Block diagram, identifying the steps of the workflow. The number of sites to be included in the initial analysis was arbitrarily set in SEMrush to 2000. Senticnet extracted the count for each pool of words classified as pertaining to one emotion. We plotted the relative occurrence of pooled emotion-related words (columns) with each site (rows) for each condition (one spreadsheet for condition) in Excel, for English sites. The fingerprint graphic format was developed for a qualitative representation of each condition emotional pattern. The variables were confronted on a statistical level (Mann–Whitney U-test) for association significance and were forwarded to SVM linear testing.

Starting point keywords were identified by the three authors who specialized in the spine (DC), knee (EK), or hip surgery (RF), as outlined in the first block in Fig. 7. Relevant internet sources were analyzed using SEMrush Competitors Research (SEMrush), a software designed for companies to run digital marketing. SEMrush can identify trends that occur within a web niche and rank performance on a content-specific base. The following parameters were evaluated for each internet source, as outlined in the second block for Fig. 7.

1. Page AS: SEMrush standard metric used to measure the overall quality of the URL and influence on SEO. The score is based on the number of backlinks, referring domains, organic search traffic, and other cases. It’s a tool to measure the impact of a webpage or domain links. Authority Score is a compound domain score that grades the overall quality of a website. The higher the score, the more assumed weight a domain or webpage backlinks could have.
2. Ref Domain: is a website that links out to another website whose backlink profile you analyze. When Google measures a domain's trust from its backlinks, the search engine weighs having a high number of referring domains. Note: the total number of referring domains that have at least one link pointing to a given URL. SEMrush only considers the domains it has seen in the last few months.
3. Backlinks are links from one website to another. Search engines like Google use backlinks as a ranking signal. Note: total number of backlinks pointing to a given URL. SEMrush only considers the backlinks it has seen in the last few months. For clarity, for a given web resource, a Backlink is a link from some other website (the referrer) to that web resource (the referent). A web resource may be (for example) a website, web page, or web directory. A backlink is a reference comparable to a citation.
4. Search Traffic: The term “search traffic” refers to the entire traffic from various visitor sources through a specific medium. Note: the amount of estimated organic traffic brought to a given URL with the keyword analyzed for a given time interval.
5. URL Keyword: The number of keywords for which a given URL ranks in search results.

### Semantic analysis—Senticnet

Content and data from URLs were converted to raw text and exported as a CSV file. The gathered data contains categories of orthopedics diseases or health conditions (back pain, hip prosthesis, knee prosthesis, etc.). Sentiment analysis (opinion mining, emotion AI) was used to extract words related to emotions. In the present study used SenticNet (https://sentic.net) a multi-disciplinary approach to opinion mining at the crossroads between affective and common-sense computing that combines semiotics, psychology, linguistics, and machine learning elements; the step is outlined as the third block in Fig. 7. Sentic computing, as opposed to statistical sentiment analysis, is a multi-disciplinary paradigm that focuses on a semantic-preserving representation of natural language concepts and sentence structure. SenticNet is based on the Hourglass of Emotions, an emotion categorization model developed to properly express the affective information associated with natural language text^56^. Using this categorization, feelings are reorganized around four independent dimensions with different levels of activation that make up the total emotional state of mind. Affective states are classified into four dimensions—Pleasantness, Attention, Sensitivity, and Attitude. Each of the four affective dimensions is characterized by six levels of activation, called "sentic levels' “, which determine the intensity of the emotion. In the *Aptitude* dimension can be found l*oathing, disgust, boredom, acceptance, trust, admiration*. In *Pleasantness*, *grief, sadness, pensiveness, serenity, joy, ecstasy*. In *Sensitivity* dimension, *terror, fear, apprehension, annoyance, anger, and rage*. Finally, in the *Attention* state, there are *amazement, surprise, distraction, interest, anticipation, and vigilance*.

BabelSenticNet^57^ is a multilingual concept-level knowledge base for sentiment analysis based on SenticNet for emotion recognition and the output returned us joy, admiration, surprise, fear, disgust, anger, sadness, and interest.

For sentiment analysis, Natural language processing (NLP) was used.

The system can then extract accurate information and insights from the papers and categorize and organize them^58^. The text has been prepared with three processes.

- Tokenization is the process of breaking down a given text into the smallest element in a sentence, termed a token. Output:” Hip”,” replacement”,” surgery”, “can”,” help”, ”relieve”
- Lemmatization, the process of discovering the normal form of an original word in the dictionary
- Part of Speech Tagging, Labeling words in a text according to their word kinds is known as Part of Speech Tagging (POS-Tag, that is noun, adjective, adverb, verb, etc.). It is a method for transforming a sentence into a list of words or tuples

Then with Sentiment analysis, a systematic identification, extraction, quantification, and study of affective states were achieved. In addition to the emotion variables, other quantities such as the number of sentences, words, content words, etc. were stored in a spreadsheet.

The documents were grouped by condition. Each partial dataset of documents from a particular group was modeled by a matrix obtained by stacking the emotional vectors. Columns pertained to emotions and rows indexed documents.

### Nonparametric statistical tests and machine learning

Comparisons were performed in the English language documents with statistical nonparametric tests (Mann–Whitney U-test, repeated three times) and by verifying health condition predictivity using different ML binary classifiers: Naive Bayes, Multi-Layer Perceptrons (MLP), Support Vector Machines (SVM) with several kernels, Decision Trees, and eXtreme Gradient Boosting (XGBoost). The classification quality results were comparable, so we concentrated on Linear SVMs, which offer optimal linear feature-space partitioning^59^, and Decision Trees, because they provide explainable results^60^. Using ML to classify the documents allowed us to find many cases with classification accuracy (assessed with a fivefold cross-validation scheme) higher than 0.90, which indicates that the document's emotional contents have peculiar patterns, in each class of a pair of health conditions, making them different and recognizable.

For evaluating performance, we computed various statistics from the confusion matrices, in particular *sensitivity (recall), specificity, precision,* and *accuracy* metrics. To also find out the optimal blend of recall and precision, we combined these two metrics in the F1 score, that is the harmonic mean of precision and recall taking both metrics into account as follows:

$$F1= 2* \frac{precision*recall}{precision+recall}$$

The harmonic mean is used instead of a simple average because the former punishes extreme values (a classifier with a precision of 1.0 and a recall of 0.0 has a simple average of 0.5 but an F1 score of 0).

This step is referenced in the last block of the diagram in Fig. 7.

## Data Availability

The datasets generated during and/or analyzed during the current study are available from the corresponding authors upon reasonable request.

## Code availability

The code used to generate results reported in the manuscript that are central to the main claims is available from the corresponding authors upon reasonable request.
